# Trabecular Reorganization in Consecutive Iliac Crest Biopsies when Switching from Bisphosphonate to Strontium Ranelate Treatment

**DOI:** 10.1371/journal.pone.0023638

**Published:** 2011-08-16

**Authors:** Björn Jobke, Andrew J. Burghardt, Burkhard Muche, Michael Hahn, Jutta Semler, Michael Amling, Sharmila Majumdar, Björn Busse

**Affiliations:** 1 Musculoskeletal Quantitative Imaging Research Group, Department of Radiology, University of California San Francisco, San Francisco, California, United States of America; 2 Institute of Radiology, Helios Klinikum Berlin-Buch, Berlin, Germany; 3 Department of Bone Metabolism and Osteology, Immanuel Hospital Berlin-Wannsee, Berlin, Germany; 4 Department of Osteology & Biomechanics, University Medical Center Hamburg-Eppendorf, Hamburg, Germany; 5 Materials Sciences Division, Lawrence Berkeley National Laboratory, University of California, Berkeley, California, United States of America; University of California, Merced, United States of America

## Abstract

**Background:**

Several agents are available to treat osteoporosis while addressing patient-specific medical needs. Individuals' residual risk to severe fracture may require changes in treatment strategy. Data at osseous cellular and microstructural levels due to a therapy switch between agents with different modes of action are rare. Our study on a series of five consecutively taken bone biopsies from an osteoporotic individual over a six-year period analyzes changes in cellular characteristics, bone microstructure and mineralization caused by a therapy switch from an antiresorptive (bisphosphonate) to a dual action bone agent (strontium ranelate).

**Methodology/Principal Findings:**

Biopsies were progressively taken from the iliac crest of a female patient. Four biopsies were taken during bisphosphonate therapy and one biopsy was taken after one year of strontium ranelate (SR) treatment. Furthermore, serum bone markers and dual x-ray absorptiometry measurements were acquired. Undecalcified histology was used to assess osteoid parameters and bone turnover. Structural indices and degree of mineralization were determined using microcomputed tomography, quantitative backscattered electron imaging, and combined energy dispersive x-ray/µ-x-ray-fluorescence microanalysis.

**Conclusions/Significance:**

Microstructural data revealed a notable increase in bone volume fraction after one year of SR treatment compared to the bisphosphonate treatment period. Indices of connectivity density, structure model index and trabecular bone pattern factor were predominantly enhanced indicating that the architectural transformation from trabecular rods to plates was responsible for the bone volume increase and less due to changes in trabecular thickness and number. Administration of SR following bisphosphonates led to a maintained mineralization profile with an uptake of strontium on the bone surface level. Reactivated osteoclasts designed tunneling, hook-like intratrabecular resorption sites. The appearance of tunneling resorption lacunae and the formation of both mini-modeling units and osteon-like structures within increased plate-like cancellous bone mass provides additional information on the mechanisms of strontium ranelate following bisphosphonate treatment, which may deserve special attention when monitoring a treatment switch.

## Introduction

Currently, several agents are available to address patients' specific medical needs when treating osteoporosis. Anti-resorptive drugs with various modes of action (e.g., bisphosphonates (BP), hormone replacement therapy, selective estrogen-receptor modulators and RANK ligand (RANKL) inhibitors slow the progression of bone loss [1], [2], [3], [4], whereas other agents stimulate bone formation (e.g., parathyroid hormone fragments) to support increases in bone mass [4], [5], [6]. Strontium Ranelate (SR) is promoted as a treatment for postmenopausal osteoporosis with a dual mechanism on bone remodeling: inhibition of osteoclastic resorption and activation of osteoblastic apposition. Its antifracture efficacy in the treatment of postmenopausal osteoporosis has been shown in previous clinical trials [7], [8], [9], [10], where Bone Mineral Density (BMD) increases under SR treatment were measured with Dual X-Ray Absorptiometry (DXA). The observed BMD gain is partially due to SR deposition in bone [9], [10], [11]. To what extent this mechanism occurs in humans as well as how it has to be considered during long-term treatment is mostly based on theoretical models and is still under investigation [12], [13], [14]. It has been shown that SR is incorporated in a dose-dependent manner into new bone (and less into pre-existing old bone) without detrimental effects on bone mineralization [11], [15], [16]. Only a few *in vivo* microstructural studies or human (histological) data have been reported about the desired effect of a positively influenced uncoupling of bone formation and bone resorption on cohorts following long-term BP treatment [11], [17]. Focusing on the previously mentioned issues, this study presents the time-associated development of cellular characteristics, serum markers, bone microstructure and mineralization caused by a therapy switch from an antiresorptive (bisphosphonate) to a dual action bone agent (strontium ranelate) in five progressively taken bone biopsies from an osteoporotic individual over a six-year period.

## Materials and Methods

Bone biopsies were obtained from the Hamburg Bone Register at the University Medical Center Hamburg-Eppendorf. The Hamburg Bone Register is a collection of diseased human bone samples representing patients covering various bone pathologies; the samples are prepared and archived in an undecalcified state. Documentation is available for each biopsy regarding the details of the patients, including their history, treatment course, and clinical complications. This resource enabled tracking and analysis of the following case of osteoporosis in a female patient over a six-year time period. The patient characteristics for this study are as follows. The patient was postmenopausal, 75 years old and had multiple vertebral fractures (Th 6–8; LS 2–4) before initiating the BP treatment. During the BP treatment, new or progressive vertebral fractures were detected with an additional reduction in height accompanied by an unchanged low BMD; thus, treatment was switched from BP to SR. The study was approved by the institutional review boards of the University of California, Berkeley & San Francisco and IDG Immanuel Diakonie Group Berlin/Brandenburg. Informed written consent was given by the patient for the study and publication of the data. Five consecutive iliac crest biopsies were obtained from the patient over a period of six years; at the same time as the biopsies, the associated hip T-Scores (comparison of the patient's BMD value to the mean BMD of the young adult healthy population) [11] and serum biomarkers were determined. Both the presence and concentration of type I collagen's crosslink peptide sequence were measured by the biomarker for carboxy-terminal collagen crosslinks (CTX). The portion of this peptide sequence that is cleaved by osteoclasts during bone resorption provides bone turnover information, where its serum levels are a proportional measure of osteoclastic activity [18]. Serum bone-specific alkaline phosphatase (bAP) was measured as a biochemical indicator of bone turnover. The bone-specific isoform of alkaline phosphatase is a tetrameric glycoprotein found on the surface of osteoblasts and can be used as an index of active bone formation [19]. Calcium and phosphorus blood samples were analyzed to differentiate between osteoporosis and other diseases associated with fracture risk and low bone mineral density [20].

**Figure 1:**
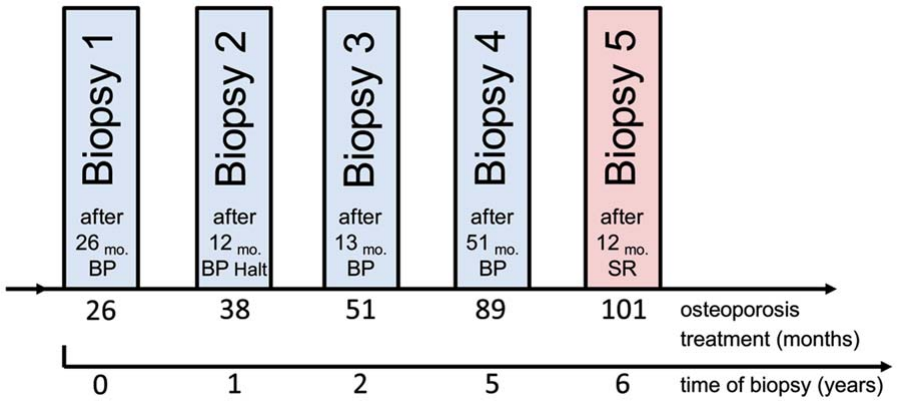
Timeline of treatment and biopsy obtainment. We investigated 5 serial iliac crest biopsies from a 75-year-old female patient over a period of 6 years. The patient was diagnosed with osteoporosis with multiple vertebral fractures and was first treated with bisphosphonates (BP) for 26 months (biopsy 1). The BP treatment was halted for 12 months until a second biopsy was obtained (biopsy 2). The BP treatment was then continued and another biopsy was taken after 13 months (biopsy 3). The antiresorptive therapy was stopped after an additional 51 months of treatment and another biopsy was taken (biopsy 4). Next, strontium ranelate was administered for 12 months (2g/d) in total before another iliac crest biopsy was conducted. BP and SR were administered according to the manufacturer's instructions and the patient additionally received a Vitamin D3 metabolite during osteoporosis treatment.

All of the bone biopsies were performed at the same clinic by one of the authors (JS, Immanuel-Hospital Berlin-Wannsee, Berlin, Germany), who has experience with over 5000 iliac crest biopsies. All of the biopsies were taken during the BP and SR treatment periods (Fig. 1), see the timeline in figure 1. The side of the pelvis that the biopsy was extracted from was alternated at each visit; biopsies taken from the same side were extracted at a similar site but at a different biopsy angle. Therefore, biopsies 1,3,5 and biopsies 2,4 were taken in an alternating caudocranial or craniocaudal direction to avoid regional overlaps and possible interference from regional acceleratory phenomenon (RAP) or other bone healing effects [6], [21]. JS used an 8-gauge Jamshidi needle with an inner diameter of 3 mm to obtain the biopsies. The biopsies meet the standard required for histomorphometry because the length of the biopsies averages 2 cm (0.79 in.). Thus, the bone volume of Jamshidi biopsies for histomorphometry equates to the bone volume of transiliacal Bordier biopsies without the limiting factor of bilateral subcortical bone heterogeneity [6], [11].

### Micro-computed Tomography (µCT)

Undecalcified and polymethylmethacrylate (PMMA) embedded biopsies were scanned with a micro-computed tomography system (µCT 40, Scanco Medical AG, Basserdorf, Switzerland) with a 12-µm isotropic nominal resolution. Application of the threshold algorithm is based on previous studies and has been described in detail [22], [23]. The bone volume fraction (BV/TV), trabecular thickness (Tb.Th.), trabecular separation (Tb.Sp.), trabecular number (Tb.N.), connectivity density (Conn.D), structure model index (SMI) and trabecular bone pattern factor (TBPf) were measured from the segmented images as described elsewhere [24]. Conn.D, SMI and TBPf are closely related to each other and provide information about the characteristics of the cancellous bone structure. Calculation of the Euler characteristics can be applied to networks to provide information regarding the number of connected entities within the structure [25], [26]. In networks of cancellous bone, the connectivity density (Conn.D) can be calculated by dividing the connectivity estimate by the volume of the sample. Basically, Conn.D provides an estimation of the number of trabecular connections per unit volume [25], [26]. The structure model index (SMI) determines the geometry of the intertrabecular network. It is based on the change in surface area with an infinitesimal increase in volume. Quantification of the SMI gives an impression of whether the trabecular structure consists of a plate-like (SMI ≈ 0) or rod-like (SMI ≈ 3) configuration [26], [27]. In cortical bone, the SMI may shift towards negative values if many concave surfaces are present [27]. The TBPf describes the ratio of intertrabecular connectivity, which was first developed to analyze histological sections in 2D [28]. The concept of TBPf is based on varying values for the convexity and/or concavity of a structure. Due to dilation, there will always be increases in bone area but increases in bone perimeter only occur with convex surfaces. Trabecular bone with many concave structures has a negative TBPf, whereas many convex structures results in a positive value (osteopenia/osteoporosis) [28]. A low TBPf value reflects a cancellous morphology that has adapted from a rod-like configuration to a plate-like configuration. Excellent correlation has been reported between the SMI and 2D-TBPf [28], [29]. The bone tissue density was determined from the original µCT grayscale images. The binary image map from the segmentation step was eroded by two voxels to remove partial volume components and to mask the grayscale data. The mean linear attenuation was calculated as the sum of the masked image divided by the number of bone voxels in the eroded mask. This value was converted to mineral density (MD) in mg HA/cm^3^ based on a phantom-derived linear calibration [22], [30].

### Undecalcified histology

Bone biopsies were sectioned to a 4-µm thickness and stained with Goldner-Masson, Giemsa and Tartrate Resistant Acid Phosphatase (TRAP). Osseous cells and osteoid indices were assessed quantitatively in accordance with the guidelines of the American Society of Bone and Mineral Research [31], [32] using histomorphometry software OSTEO II (Bioquant, Nashville, TN, USA). The ratio of the osteoid surface to the bone surface (OS/BS,%), the ratio of the osteoblast surface to the bone surface (Ob.S/BS,%), the ratio of the number of osteoblasts to the bone surface (N.Ob/BS, #/mm) and the ratio of the number of osteoclasts to the bone surface (N.Oc/BS, #/mm) were determined. Bone turnover was classified by analyzing serum markers (bAP and CTX) [33], [34], [35], [36], [37], [38], [39] and osteoid indices [40], [41], [42], [43], [44], which were compared to reference values from the literature for naïve and treated patients. Furthermore, visible bone remodeling units were evaluated [45] as an additional supportive measurement indicating bone turnover information and were based on the following histological criteria. The total number of osteoblasts and osteoclasts were counted at individual bone remodeling units (magnification 40x) and divided by the total number of assessed fields of view (approx. 4–6). ‘*Low turnover*’ is attributed to bone tissue under low endosteal remodeling showing <1 bone remodeling unit with cubic-shaped osteoblasts and a maximum of one osteoclast per field of view (magnification 40x) in the underlying histological section. *‘Normal turnover’* is attributed to bone tissue with maintained endosteal remodeling showing 1–2 bone remodeling units with cubic-shaped osteoblasts and 2–4 single osteoclasts per field of view (magnification 40x). *‘High turnover’* is attributed to bone tissue with both increased endosteal remodeling and osteoclastic resorption showing >2 bone remodeling units with prominent seams of osteoblasts and >4 groups of osteoclasts per field of view (magnification 40x) in the underlying histological section. Polarized light was used to observe collagen birefringence and lamellar orientation [46], [47].

### Bone mineral density distribution and microanalysis

The bone mineral density distribution was measured on plastic-embedded cross-sections of iliac crest biopsies with coplanar, polished and carbon-coated surfaces using quantitative backscattered electron imaging (qBEI) [11], [48], [49], [50]. The scanning electron microscope (LEO 435 VP, LEO Electron Microscopy Ltd., Cambridge, England) was operated at 15 kV and 665 pA at a constant working distance (BSE Detector, Type 202, K.E. Developments Ltd., Cambridge, England). The pixel size was 3 ìm, as recommended by Roschger et al. [50]. Synthetic hydroxyapatite (HA) samples were used to create a calibration curve. These HA samples (DOT Medical Solutions, Rostock, Germany) contained different Ca/P ratios, which were determined with energy dispersive X-ray analysis (DX-4, EDAX, Mahwah, NJ) and qBEI. A highly linear relationship between the gray values of backscattered signal intensities and the calcium content of each sample has been reported previously by other authors [49], [51], enabling calibration of the method. The generated mineralization profile (gray value histogram) reflects the degree of mineralization in the cross-sectioned bone.

Strontium deposition in mineralized bone tissue was measured using µXRF (Micro-X-ray Fluorescence Analysis; IMOXS, IFG, Germany) in combination with EDX (Energy Dispersive X-ray Analysis); EDAX DX-4, USA). An XRF-module with capillary zoom optics enables x-ray fluorescence analysis in the SEM with a high sensitivity. The advantage of this method is the high spatial resolution [11], [52]. The detection limit ranges between 1 and 0.01 weight percent (wt%) due to electron-induced Bremsstrahlung background. The strontium (Sr) content in the bone biopsies was evaluated in wt% via software that enabled combined EDX/µXRF quantification (IMOXS Quant 2.10, IFG, Berlin, Germany).

## Results

### Clinical data

**Figure 2:**
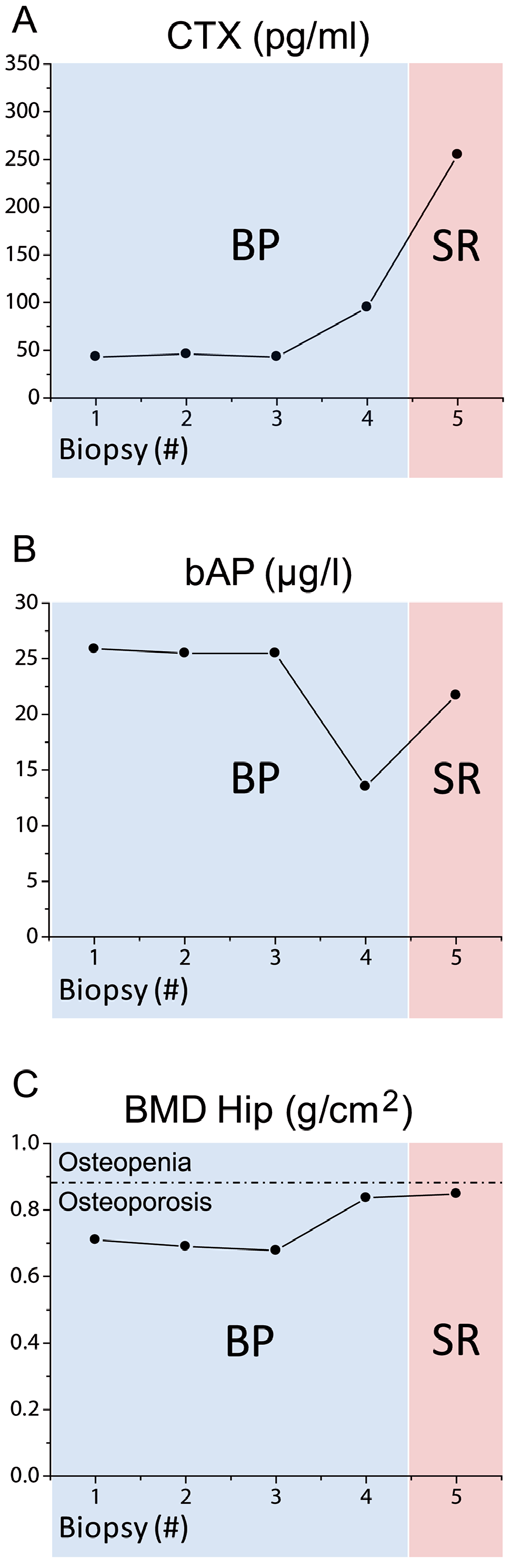
Clinical data. **A**) C-terminal cross-linking telopeptides of type I collagen (CTX) showed very low levels under antiresorptive therapy and rose to normal levels after SR treatment was initiated. **B**) The bone specific alkaline phosphatase (bAP) level varied under BP treatment from 25.9 to 13.5 µg/l. After the treatment was switched to SR, the bAP level rose again to 21.7 µg/l. **C**) DXA measurements showed only marginal changes in bone mineral density at the femoral neck following both the BP and SR treatments.

C-terminal cross-linking telopeptides of type I collagen (CTX) were consistently at very low levels (43–95 pg/ml) under antiresorptive therapy (Fig. 2A) in comparison to the range of mean CTX values (331–562 pg/ml) in the literature for naïve postmenopausal women [33], [38]; after antiresorptive treatment, the CTX values may range at approx. a quarter of the baseline [34], [35]. CTX rose to moderate levels (255 pg/ml) [33], [38] after the SR treatment was initiated (Fig. 2A). At the same time, the bone specific alkaline phosphatase (bAP) level rose from 13.5 to 21.7 µg/l (Fig. 2B) in comparison to the range of mean bAP values (13.6–17.3 µg/l) from the literature for naïve postmenopausal woman [33], [36], which may decrease by approx. 45% during bisphosphonate treatment [37], [39]. Calcium/phosphorus levels were in the normal range [53]. During BP treatment, new or progressive vertebral fractures (Th 7, 8, 12; LS 2, 3) were determined with an additional 6-cm reduction in height. DXA measurements showed only minor changes in the BMD at the femoral neck following BP treatment and following SR treatment (Fig. 2C). The T-score at the lumbar spine increased by 0.3 T-score points to −3.5, but its interpretation was very limited due to multiple fractures. No DXA density correction factor related to the period of strontium ranelate treatment was applied.

### µCT tomography

**Figure 3:**
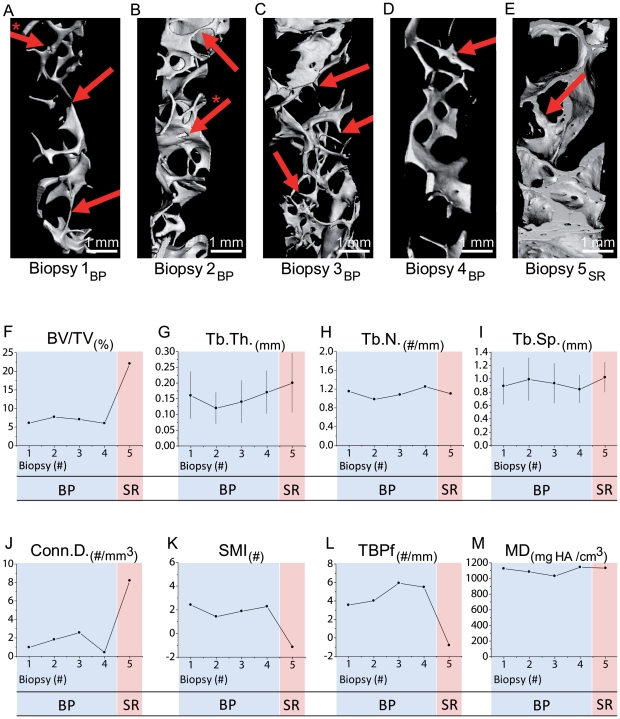
µCT reconstructions of biopsies and structural indices. **A**) µCT-3D reconstruction of the complete bone core (biopsy 1). Rod-like structures with a microcallus formation (*→) are evident in the trabecular architecture. Some rods are likely to be perforated (→). **B**) µCT reconstruction of biopsy 2. Bone core with a combination of plate- and rod-like architecture. Note the thinning of the connecting rods (→) and reticulate perforation sites on a plate (*→). **C**) µCT reconstruction of biopsy 3. The trabecular architecture of this bone core presents an irregular composition of fine rod-like elements with some small plates. Several dead-ending rods are visible (→). **D**) µCT reconstruction of biopsy 4. The trabecular architecture in this iliac crest sample is almost completely deteriorated. Another trabecular fracture of a rod leading to microcallus formation is visible (→). Microcallus formations are bulky nodes with an irregular oftentimes clefted structure. **E**) µCT reconstruction of the biopsy after one year of SR treatment showing thick plate-like structures containing multiple intratrabecular ‘tunnels’. Although a very dense architecture is evident in the structure, big gaps of lost connectivity are also visible. Bulky microcallus formation (→). **F**–**L**) Changes in the trabecular network emphasized by structural indices evaluated through assessment of 3D µCT reconstructions. The dots represent mean values, and the error bars represent standard deviations of the mean. **M**) Mean density measured by µCT showed already high values during BP treatment that can be traced back to low bone turnover. The mean density after 12 months of SR remained almost unchanged suggesting maintained mineralization during a normal bone turnover situation.

The bone volume fraction, structural parameters and bone tissue density data from all five biopsies collected by µCT are shown in figure 3. In comparison with all biopsies after BP treatment (Biopsy 1, 2, 3 and 4), the trabecular architecture after SR administration (Biopsy 5) changed notably (Fig. 3A–E). The trabecular architecture in biopsies 1–4 (Fig. 3A–D) consisted mainly of thin rod-like structures or plates connected by very thin rods. In biopsy 5 (Fig. 3E), a high increase in BV/TV (22% vs. 6%) along with an increase in Tb.Th. (0.2 vs. 0.17) was observed (Fig. 3F,G). The 3D reconstruction shows thick, plate-like structures that seemed to be connected mainly by plates and a small number of rods, which represents a small reduction in trabecular number and a very high connectivity density (Conn.D.: 8.2) (Fig. 3H–J). The SMI (Structural Model Index) and trabecular bone pattern factor (TBPf) presented had improved values (SMI; −0,81 vs. 5,49 and TBPf; −1,15 vs. 2,27) (Fig. 3K,L). Reconstruction of biopsies 1,4 and 5 showed small microcallus formations as a result of biomechanical instability and thus trabecular fracture (Fig. 3A,D,E). The mean density measured by µCT in milligrams of hydroxyapatite per bone tissue volume had already reached a high value of 1126 mg HA/cm^3^ after 26 months of BP treatment (Biopsy 1), dropped after cessation of antiresorptive treatment to 1085 mgHA/cm^3^ (Biopsy 2) and increased again to 1144 mgHA/cm^3^ under a status of reduced bone turnover (Biopsy 4). The mean density after 12 months SR remained almost unchanged at 1133 mg HA/cm^3^ (Biopsy 5) during a normal bone turnover situation (Fig. 3M).

**Figure 4:**
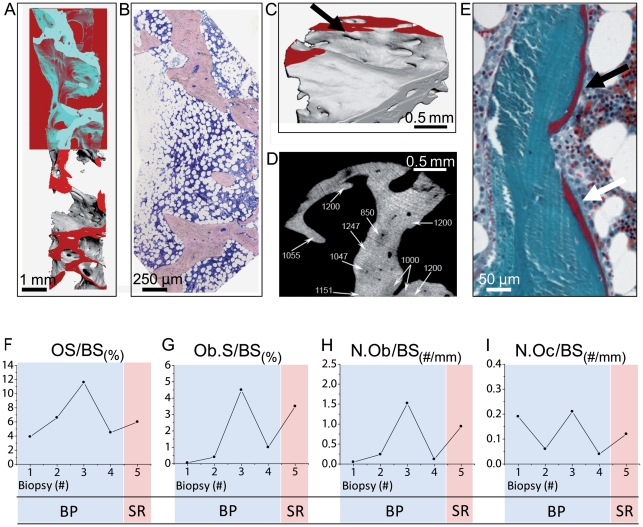
Reorganization pattern, µCT mean density and 2D histomorphometry. **A**) µCT reconstruction of biopsy 5. Note the coarse structure with surface irregularities and the occurrence of an intratrabecular ‘tunnel’. The area with the dark-colored background demonstrates the 2D section plane as it can be seen on the histology section in panel B. **B**) Congruent histology section following the µCT slice in panel A. Note the irregular trabecular structure with varying trabecular diameter. The thickened trabecular architecture indicates intratrabecular resorption sites filled with fibrovascular tissue (mineralized bone  =  light purple; Giemsa, 40x). **C**) A high magnification of the µCT reconstruction of biopsy 5 indicates the irregular trabecular surface with multiple hook-like, tunneling and sometimes perforating resorption lacunae. Virtual serial sections can show that all intratrabecular ‘tunnels’ are connected at some point and have openings to the trabecular surface. Small humps due to bone formation (mini-modeling) arise from the trabecular surface (→). **D**) Axial µCT section of biopsy 5: Distribution of local mineral densities (mg HA/cm^3^) represented by different gray levels. Due to bone formation, a low mineral content is detected in the proximity of new osteons, whereas the interstitial regions show a higher mineral content due to an older tissue age. **E**) Two (re-)modeling sites with distinct osteoid seems are visible. The osteoid seam on the trabecular surface at the bottom (white arrow) is covered with some cubic-shaped osteoblasts that become flattened to the lower image border. Non-active endosteal appostion is evident on the top of the image (black arrow) without prior resorption (modeling) as flat osteoblasts or lining cells cover osteoid (mineralized bone  =  green, osteoid  =  red; Goldner-Masson, 400x). **F–I**) Osteoid and cellular indices determined by 2D histomorphometry point to a low bone turnover situation at the time of biopsies 1, 2 and 4. Normal bone turnover, leading to an increase in osteoid apposition and thus increasing osteoid surface and volume appeared only after 50 months of BP treatment and after the treatment was switched to strontium ranelate. Osteoid indices did not indicate any signs of hyperosteoidosis considering the reference ranges reported by Lips et al. [73].

In contrast to flat, reticulate, perforating resorption cavities in biopsies during BP treatment (Fig. 3A–D), multiple deep, hook-like [15] resorption patterns were visible on the surface and on sections of biopsy 5 (Fig. 3E,4A). Small humps of endosteal bone (peritrabecular surfaces) were detected in µCT reconstructions and histology (Fig. 4B–E). Peak densities measured in the cancellous bone ranged between 1200–1300 mg HA/cm^3^ (Fig. 4D). A maximum value of 1400–1500 mg HA/cm^3^ was detected in a microcallus formation in biopsy 5 (Fig. 3E). The lowest values in recently mineralized modeling sites ranged between 650 to 850 mg HA/cm^3^ (Fig. 4D).

### Undecalcified histology

**Figure 5:**
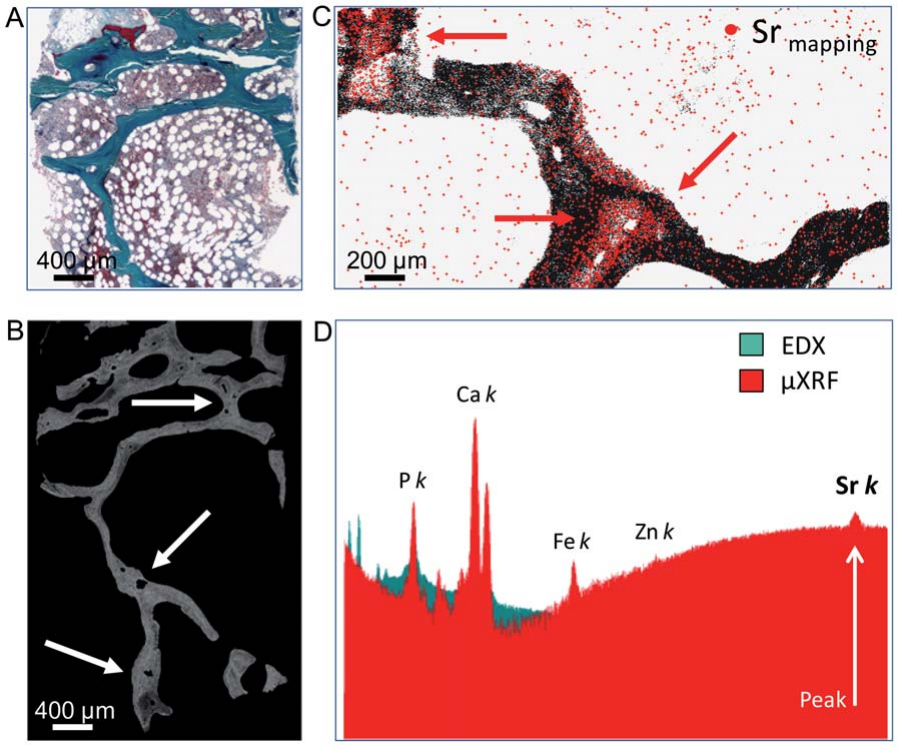
Bone mineral density distribution and microanalysis. **A**) Histology of the cancellous bone area after strontium ranelate treatment. Intratrabecular cavities and thin osteoid seams are visible (mineralized bone  =  green, Goldner-Masson, 40x). **B**) Quantitative backscattered image of the selected area in panel A. Intratrabecular resoption sites showed osteon-like structures (→). The gray level distribution reflects the mineralization density, which varied between new and old bone. Newly formed osteon-like structures showed dark-gray pixels in comparison to older bright-gray bone packets. **C**) Microanalysis mapping showed that increased strontium deposition (red dots) appeared predominantly around newly formed osteon-like structures (→) as well as in modeling sites on endosteal surfaces (→). **D**) Combined EDX/µXRF microanalysis spectra demonstrated a deposition of 1.63 Wt% strontium (peak) in the mineralized tissue after SR treatment.

Following 12 months of SR administration, individual trabeculae were thicker as expected in healthy women of that age group [54], [55] and presented a somewhat irregular connectivity (Fig. 4B,E). Woven bone was not observed in any of the biopsies. However, following strontium ranelate treatment, several thick trabeculae had deep intratrabecular resorption cavities filled with fibrovascular tissue (Fig. 4B). Osteon-like structures in the trabecular bone were seen frequently (Fig. 4B). Seams of osteoid only partially covered with osteoblasts were occasionally present in the form of endosteal humps (Fig. 4C,E) on top of straight cement lines, which suggests that endosteal apposition took place without prior resorption (modeling). A genuine fibro-osteoclasia was not visible. Remodeling indices (Fig. 4G–J) determined by 2D histomorphometry (Fig. 5A) provide histological bone turnover information. Normal mean values for OS/BS have been reported between 9.51% and 35% for female cohorts [40], [41], [42], whereas a bisphosphonate treatment regime is known to decrease OS/BS mean values to within a range of 1.02% to 3%, in accordance with low bone turnover [43], [44] (Fig 4G).

### Bone mineral density distribution and microanalysis

Backscattered signal intensities reflected a maintained mean hard tissue mineralization (Fig. 5B). Combined EDX/µXRF microanalysis revealed a deposition of 1.63 Wt% strontium in the mineralized tissue after SR treatment (Fig. 5C,D). Lower mineralized regions (dark gray values) showed increased strontium content and appeared more commonly around osteon-like structures (Fig. 5B,C) but were also present in modeling sites on endosteal surfaces (peritrabecular surfaces) (Fig. 5C). Before treatment, the strontium content ranged at the detection limit of the method.

## Discussion

One year of SR administration following long-term bisphosphonate treatment revealed a notable increase in bone volume fraction. This structural change reflected more of a transformation of trabecular rods to plates than an increase in trabecular thickness and number because indices of connectivity density, structure model index and trabecular bone pattern factor were predominantly enhanced. The degree of mineralization was maintained although strontium deposition occurred on the bone surface level. No mineralization defects (e.g. entrapped osteoid) or accumulation of osteoid were detected. The appearance of tunneling resorption lacunae, the new formation of osteon-like entities and mini-modeling of endosteal humps in trabecular structures reflected a novel particularity after treatment switch accompanied by an increase in cancellous bone mass.

The enhancement in bone volume fraction following SR treatment, which was evaluated by micro-tomography, was not equally represented by increases in trabecular thickness or trabecular number. The characteristics of the trabecular architecture changed to a partly disorganized and somewhat compact network without separated trabeculae; thus, the number of individual trabeculae decreased but the connectivity density increased. The structure model index (SMI) and trabecular bone pattern factor (TBPf) were both negative, which may indicate the formation of very dense, plate-like structures or many concave structures created by tunneling resorption [29], [56]. The discovered reorganization mechanism also raises the question of whether this could eventually lead to an increase in ‘new’ trabeculae. Compared to normal trabecular thickness values obtained from histomorphometric studies [54], [55], an unusual thickness of 300–400 µm was measured for many plate-like structures. Many of these thick trabeculae included newly formed osteon-like structures, which are characteristically found in the cortex around Haversian canals [57]. µCT observations revealed that a majority of these canals or tunnels create a complex, interconnected system as can be seen in the cortex between Haversian and Volkmann canals. The relevancy of its development in cancellous bone remains uncertain. It can only be speculated whether this new intratrabecular canal system that has developed outside of the cortex maintains blood supply to the osteocyte network in the inner regions of unusually thick trabeculae [58]. Inta-trabecular osteons may act to ensure adequate diffusion of nutrients to, and waste products from, osteocytes by limiting the maximum distance between the osteocytes and the bone surface [59]. In particular, thin nonmineralized pericellular zones between osteocytes and their surrounding bone allow a strain-derived flow of interstitial fluid over the osteocytes' surfaces that is important for the osteocytes' health because it facilitates the exchange of nutrients and waste products between the haversian channel and the osteocyte network [60], [61]. Although not investigated in this study, a dramatic change in the cancellous structure from a plate-like to rod-like shape with aging and osteoporosis has been reported and results in a decrease in mechanical competence [62], [63]. The measured reversal of the bone structure due to changes in treatment may therefore improve the bone's elastic modulus [64]. There are several possible factors that may account for the dramatic increase in bone volume. Because the trabecular thickness only slightly increased by a mean of 0.03 mm, which only explains about one third of the gain in bone volume, a possible explanation was found in the reorganization pattern. Endosteal appostion on the trabecular surfaces without prior resorption (modeling) was found in the biopsy after SR treatment. These small modeling sites, which are also known as *mini-modeling* [65], [66], thicken and rearrange the remaining trabeculae to best adapt to functional demands. Multiple long and deep, hook-like resorption cavities, which are frequently seen and reported in renal osteopathies (e.g. ROD type IIIb) [67], were observed. Similar resorption sites were first described by Woods (1972) and later quantified by Sato (1981) under conditions of osteoidosis and increased PTH serum levels [68], [69]. The cause was suspected to be multifactorial, including maintenance of calcium homeostasis through a compensatory increase in resorption depth in osteomalacic situations and PTH-induced overstimulation of osteoclasts. Low calcium or abnormal PTH serum levels were not found in our patient but could have played a role in the early stages of SR-induced bone formation that created a temporary calcium deficit and a need for increased bone resorption. The SR treatment stimulated bone turnover, which was demonstrated by moderate serum bone markers (bAP, CTX), and indicated increased bone formation (endosteal humps with osteoblast coverage and newly formed bone packets with an accumulation of strontium). An uncoupling with a pronounced suppression of osteoclastic resorption was not present histologically or biochemically after 12 months of SR administration. However, a complete uncoupling was not expected because it is known that an osteoclast-derived stimulus is required for normal bone remodeling involving bone formation. In this connection, osteoclastic resorption re-activated by strontium ranelate's mode of action may account not only for the improvement in bone mass and structural integrity but also for the maintenance of calcium metabolism.

Previous reports have shown that strontium is predominantly incorporated into new, and to a lesser degree, old bone in a dose-dependent matter without altering the degree of bone mineralization [11], [15], [16]. Based on theoretical models, other groups report that calcium substitution by strontium at the crystal level and its higher attenuation of x-rays could account for up to 10% of the total bone mineral density as measured by DXA, depending on the time of SR administration [12], [14], [70]. Clinical BMD scores acquired from the hip with DXA did not change significantly in our patient. The only assumption the authors could make from these results was that newly formed bone with a low mineral content was not able to offset the deterioration of the trabecular architecture in the femoral neck that may have irreversibly progressed before SR treatment was initiated. µCT uses a similar X-ray (fan beam) technique and is potentially influenced by the degree of attenuation. The strontium content in the bone after 12 months of treatment in the investigated patient clearly rose significantly to 1.63 Wt%. Despite the fact that we observed some areas with an unusually high degree of mineralization (>1200 mg HA/cm^3^), the average mean tissue density (µCT) after SR administration did not change significantly. This finding might be due to the fact that the tissue density in biopsy 4 (1133 mg HA/cm^3^) was already high and had reached a maximum saturation point. Therefore, tissue mineralization would not have a major impact on bone mineral density measurements.

This study has several limitations. First, the heterogeneity of the trabecular microstructure in different sites of the skeleton may have an effect on the accuracy of the measured structural indices [48], [71], [72]. Second, because more than one biopsy was obtained on each side of the Ilium, there remains the possibility that a later biopsy will overlap in parts with the location of a prior biopsy and include regions of the Ilium that healed following the earlier biopsy. Third, the study of five biopsies is limited to only one individual.

In this study, analyses of progressively obtained iliac crest bone biopsies over six years of osteoporosis treatment highlight remarkable changes in cancellous architecture, which are reinforced by a switch from an antiresorptive agent to a dual action bone agent. The clinical and microstructural information gained by an unusually high number of bone biopsies demonstrated treatment-associated responses in a novel case. These insights may support the understanding of reorganization mechanisms due to alternating osteoporosis treatments. Clinical bone markers showed that strontium ranelate stimulated bone formation and simultaneously bone resorption despite prior long-term antiresorptive treatment. In this case, administration of strontium ranelate for one year led to remarkable changes in trabecular structure with an impressive gain in bone volume fraction at the iliac crest, which may contribute to changes in bone quality.
